# Retrosternal gentamicin-collagen sponge does not reduce the incidence of mediastinitis in cardiac surgery

**DOI:** 10.1186/1749-8090-8-S1-O116

**Published:** 2013-09-11

**Authors:** T Madej, M Szlapka, F Neumann, K Plötze, K Matschke, T Waldow

**Affiliations:** 1 Department of Cardiac Surgery, Dresden Heart Centre, Germany

## Background

Reduction of poststernotomy mediastinitis (PSM) remains a challenge for cardiac surgery. This prospective registry study was conducted to evaluate the effect of implantable gentamicin - collagen sponge used retrosternal on the incidence of mediastinitis after cardiac surgical procedures via median sternotomy.

## Methods

Between September 2011 and June 2012 a total of 1372 consecutive patients underwent cardiac surgery at our institution. Of these patients, 1048 underwent elective procedures via median sternotomy, met inclusion criteria and were enrolled into the study. The complete cohort was prospectively divided into two registries. In treatment registry (543 patients), a collagen sponge containing 130 mg gentamicin was implanted retrosternal before sternal closure. In control registry 2 (505 patients) the sponge was not used. Primary endpoint was freedom from mediastinitis according to CDC criteria on the 30th postoperative day. Secondary endpoint was freedom from surgical site infection of any kind.

## Results

Both registries were matched in baseline characteristics as well as known risk factors. Most common isolated pathogens causing a surgical site infection were Gram-positive species - S. aureus and S. epidermidis. No MRSA was isolated as a cause of mediastinitis. Incidence of PSM was 2.7% in treatment registry and 3,4% in control registry and was not significantly different. In reference to secondary end-point, no significance was seen, either.

## Conclusion

Retrosternal application of 130 mg gentamicin - collagen sponge exerts no influence on incidence of mediastinitis in elective adult cardiac surgery with median sternotomy.

